# A feasibility study of altered spatial distribution of losses induced by eddy currents in body composition analysis

**DOI:** 10.1186/1475-925X-9-65

**Published:** 2010-11-04

**Authors:** Kim H Blomqvist, Raimo E Sepponen

**Affiliations:** 1 Department of Electronics, Aalto University, PO BOX 13340, 00076 Aalto, Finland

## Abstract

**Background:**

Tomographic imaging has revealed that the body mass index does not give a reliable state of overall fitness. However, high measurement costs make the tomographic imaging unsuitable for large scale studies or repeated individual use. This paper reports an experimental investigation of a new electromagnetic method and its feasibility for assessing body composition. The method is called body electrical loss analysis (BELA).

**Methods:**

The BELA method uses a high-Q parallel resonant circuit to produce a time-varying magnetic field. The Q of the resonator changes when the sample is placed in its coil. This is caused by induced eddy currents in the sample. The new idea in the BELA method is the altered spatial distribution of the electrical losses generated by these currents. The distribution of losses is varied using different excitation frequencies. The feasibility of the method was tested using simplified phantoms. Two of these phantoms were rough estimations of human torso. One had fat in the middle of its volume and saline solution in the outer shell volume. The other had reversed conductivity distributions. The phantoms were placed in the resonator and the change in the losses was measured. Five different excitation frequencies from 100 kHz to 200 kHz were used.

**Results:**

The rate of loss as a function of frequency was observed to be approximately three times larger for a phantom with fat in the middle of its volume than for one with fat in its outer shell volume.

**Conclusions:**

At higher frequencies the major signal contribution can be shifted toward outer shell volume. This enables probing the conductivity distribution of the subject by weighting outer structural components. The authors expect that the loss changing rate over frequency can be a potential index for body composition analysis.

## Background

Central (visceral) obesity has an important role in the development of type 2 diabetes [1]. One recent study has also found that fat collection around the heart and aorta and within the liver is clearly associated with decreased heart functions [2]. The same study also shows once again that body mass index (BMI) has limitations as a health metric.

Tomographic imaging is an accurate and reliable method to measure the visceral fat area (VFA), but it is typically slow and expensive. Thus it is not suitable for evaluating large groups of individuals. Moreover, X-ray computed tomography (CT) exposes patients to radiation. Therefore there is a need for a simple and cost-effective method to measure visceral fat accumulation [3,4].

Waist circumference has been used as an alternative index because of its simplicity and reasonable correlation with CT. However, it is desired to replace it by a more direct measurement which would not be affected by the subcutaneous and the fat free volumes. Abdominal bioelectrical impedance analysis has been proposed as a suitable low-cost method for assessing VFA, but in spite of its potential, further exploration of the method is still needed [5].

This paper introduces a body electrical loss analysis (BELA) method for body composition analysis. BELA is an electromagnetic method, where the object is placed in a time-varying magnetic field. The object set in the magnetic field perturbs the field, and the magnitude of this perturbation is measured. The first work related to measurements of the perturbation of biological tissue in a magnetic field was carried out by Tarjan and McFee [6]. Later on, magnetic fields have been used in the assessment of the composition of the human body, such as in the total body electrical conductivity (TOBEC) method [7]. TOBEC operated on the principle that the impedance of a solenoidal coil is changed when a subject is placed into a coil. In the last decade much research has been done towards the realisation of an imaging method based on magnetic induction [8-10]. In magnetic induction tomography the excitation coils are used to induce eddy currents in the subject, and the magnetic field from these is then detected by sensing coils.

The device employed in the present study is a feasibility model designed specially for the studies of the abdominal region of an adult human subject. The sensitivity of the method to the eddy current losses arising from the sample was validated with simplified phantom tests and the results are shown below.

## Methods

**Figure 1:**
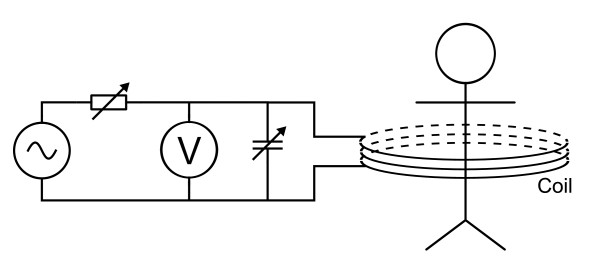
**BELA method**. Resonant circuit matched to voltage divider circuit. Eddy currents induced in the torso are observed as a voltage change in the voltage divider.

BELA method uses a high-Q parallel resonant circuit to produce an oscillating magnetic field. The resonator is matched to a voltage divider circuit, as shown in Figure 1. When a conductive object is placed into the coil of the resonant circuit an internal electric field **E **caused by the time-varying magnetic field **B **induces eddy currents in the object [11] and power is dissipated. This appears as reduced Q of the coil (resonant circuit) and reduced impedance *Z *of the coil, which for a high-Q [12] unloaded coil are given by

(1) $$Q=\frac{\omega L}{R_{\text{c}}}$$

(2) $$Z=\frac{X^{2}}{R_{\text{c}}}-\text{iX}$$

respectively, with an angular frequency *ω *= 2π*f*_unloaded _and where *f*_unloaded _is the resonance frequency of the unloaded coil, *L *is the inductance of the coil, *X *= 1/*ωC *= *ωL*, and i = $\sqrt{-1}$. *R_c _*represents the losses of the coil accounting for the ohmic resistance in series with the inductance and the losses of the tuning capacitor [13]. Further-more, from equation 2 it should be noticed that |*Z*| ≈ *X*^2 ^/ *R_c _*for a resonant circuit with high Q and large *L*. The quality factor of the loaded coil is given by

(3) $$Q_{\text{L}}=\frac{\omega_{\text{loaded}}L}{R_{\text{c}}+R_{\text{L}}}$$

where *ω*_loaded _= 2π*f*_loaded _and *R*_L _is the equivalent sample resistance which loads the coil [13].

The losses generated by eddy currents in the human body are related to the body electrolytes. Since lean tissue is effectively over ten times more conductive electrically than fat or bone [14], it produces significantly more losses than fat. The conductivity of adipose tissue is almost constant over the frequency range from 1 kHz to 1 MHz, whereas the conductivity of muscle tissue alters a little bit, especially if the electric field is across the fibre. For sinusoidal steady-state electromagnetic fields, the power dissipated in an infinitesimal volume element Δ*v *of a material is given by

(4) $$P=\sigma_{\text{eff}}E_{\text{rms}}^{2}\Delta v$$

where $\sigma_{\text{eff}}=\omega \epsilon_{0}{\epsilon^{\prime }}_{\text{r}}\mathrm{tan\delta}$ is the effective conductivity (low frequency approximation) and *E*_rms _is the rms value of the electric field **E **[15].

**Figure 2:**
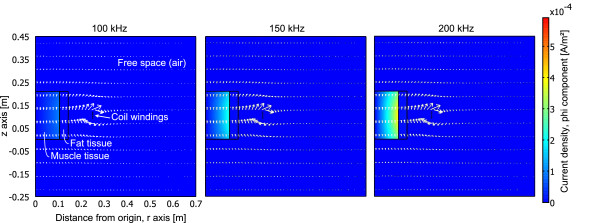
**Induced eddy currents in test phantom, case 1**. Phi component of total current density [A/m^2^] in test phantom with fat in outer shell volume at excitation frequencies of 100 kHz, 150 kHz and 200 kHz. The arrows point in the direction of the magnetic flux.

**Figure 3:**
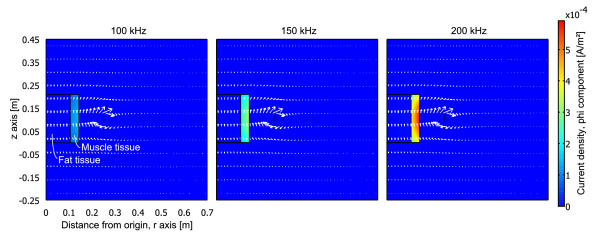
**Induced eddy currents in test phantom, case 2**. Phi component of total current density [A/m^2^] in test phantom with fat in the middle of its volume at excitation frequencies of 100 kHz, 150 kHz and 200 kHz. The arrows point in the direction of the magnetic flux.

The purpose of the BELA method is to probe the conductivity distribution of the subject, by weighting outer structural components using multiple frequencies. The **B **applied parallel to the z-axis of the uniform cylindrical sample induces an internal electric field in the sample which magnitude at cross-sectional layer is *E*_int _= *ωBr*/2, where *r *is the distance from the z-axis to the point at which the induced **E **field is evaluated and *B *is the magnitude of the magnetic field [16]. The loading effect caused by the sample placed in the coil, is heavily dependent on the conductivity of the outer shell volume of the sample. Thus the loss changing rate over frequency for a sample that has a more conductive outer shell volume than its interior, is higher than it would be for a sample with reversed conductivity distributions. This is due to the fact that at higher frequencies the induced eddy currents get stronger and, because of the skin effect phenomenon, are more concentrated on the outer radius of the sample. Figures 2 and 3 illustrate this phenomenon in a cylindrical coordinate system for two test phantoms. The volume, where the induced currents are minimal, mimics fat tissue with a conductivity of *σ *= 0.04 S/m and a relative permittivity of ϵ_*r *_= 1 × 10^2^. The more conductive volume mimics muscle tissue (*σ *= 0.75 S/m, ϵ_*r *_= 4 × 10^3^). The simulations were performed with COMSOL Multiphysics 3.5a by using the time-harmonic analysis of azimuthal induction currents (vector potential) of an AC/DC Module. The current flowing in the coil windings was set to 7 mA, which is the case at the highest resonant frequency used in this study. The boundary conditions are magnetic insulation for the free space, continuity for the phantom and axial symmetry where r = 0.

The altered spatial distribution of the losses induced by eddy currents in the sample is a new approach to body composition analysis, and it is how the BELA method differs from the former non-imaging electromagnetic methods, such as TOBEC [17-19], electromagnetic resonance [20], and tissue resonant impedance monitor [21].

### Sensor and instrumentation

**Figure 4:**
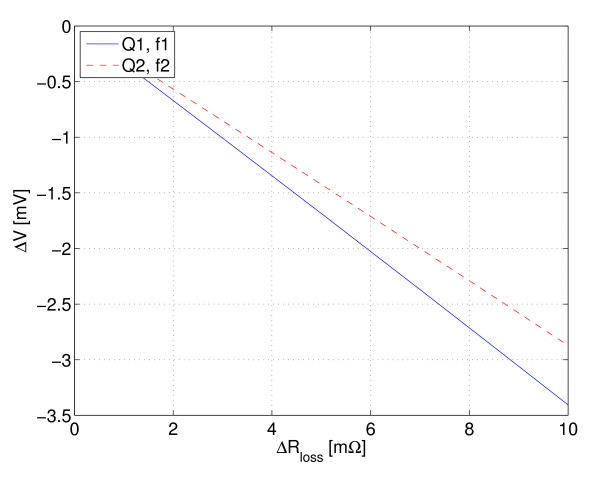
**Example sensor response**. Equation 5 plotted for two different excitation frequencies over the region of Δ*R*_loss _observed in practice. *V*_0 _= 1 V, *Q*_1 _= 95, *Q*_2 _= 145, *f*_1 _= 103 kHz and *f*_2 _= 186 kHz (refer to Figure 12).

The coil was constructed from litz wire wounded to a form of rectangular helical coil (50 cm × 70 cm with 8 turns on a wooden frame and with an inductance of about 110 *μ*H). The final sensor consists of a series resistor connected to a resonant circuit to form a voltage divider. By using equations 1 and 2 one can find that the voltage change Δ*V *in the voltage divider produced by the change in loss resistance Δ*R*_loss _= *R*_L _is approximately

(5) $$\Delta V\approx V_{0}{\left(6-4\frac{\omega L}{Q}\frac{1}{\Delta R_{l\text{oss}}}\right)}^{-1}$$

where *V*_0 _is the amplitude of the time-varying voltage applied across the voltage divider. The approximation *Z *≈ *X*^2^*/R *was used. As can be seen from this equation, the sensitivity of the sensor is not flat over the frequency bandwidth. Although a reasonably flat Q could be achieved over the bandwidth, there is still the frequency dependency which affects the impedance level of the voltage divider. However, in the region of Δ*R*_loss _observed in practice, the response is very linear, as shown in Figure 4. A compensation factor *k *≈ *Q*_1_*f*_2_*/Q*_2_*f*_1 _between two resonance frequencies can be found and the measured voltages at different resonance frequencies can be made comparable.

**Figure 5:**
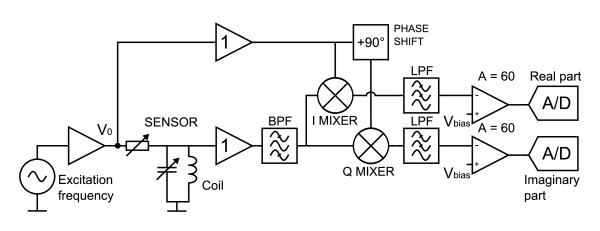
**Block diagram of the instrumentation**. The phase-sensitive detector (also known as a lock-in amplifier) makes extraction of a signal with a known carrier wave in a noisy environment possible. Buffer amplifiers are used to isolate the sensor from the detector part of the electronics.

The instrumentation is shown in the block diagram of Figure 5. The resonant circuit is driven from a waveform generator that supports sweeping to locate the resonance frequency. Both the real and imaginary parts of the voltage across the resonant circuit are measured. The value of the voltage for the empty coil is subtracted from that of the loaded coil by setting the voltage *V*_bias _of a difference amplifier. The voltage difference (*V*_loaded _- *V*_empty_) is amplified by 60 to get higher sensitivity.

## Experiments

### Phantom test

The feasibility of the BELA method was tested with simplified phantoms. Four phantoms with different conductivity distributions were placed in the coil and the voltage change across the coil was measured. The measurement was performed as a trial test at five different resonance frequencies. The frequency was changed by switching a tuning capacitor and matching resistor. The number of consecutive sweeps was 16 (average factor) and the amplitude of the excitation frequency was set to 1 V. After the resonance was found and the span narrowed, the frequency increments of the sweep were 2 Hz.

**Figure 6:**
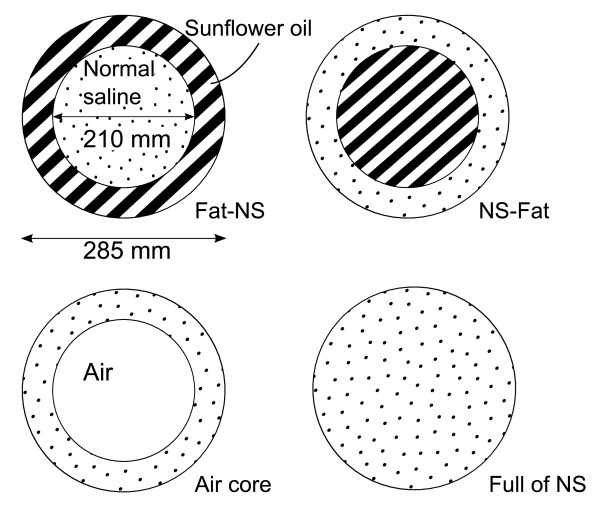
**Phantoms**. Physical dimensions and the fillings of the phantoms used. NS means normal saline (NaCl 0.9%).

Every phantom was made from two straight-walled cylindrical buckets, one with a capacity of 10 litres and the other of 5 litres. The smaller bucket was placed inside the bigger one to get two separate volumes, an outer shell volume and a middle-part volume. Two of the phantoms were filled with a sun flower oil and saline solution, as shown in Figure 6. These two phantoms are rough estimations of the human torso where one has only subcutaneous fat and the other visceral fat without subcutaneous fat. Rather similar models were used in [4]. Although the human torso has more of an elliptical cross-section than a cylindrical one, the use of a phantom with an elliptical cross section was not considered to be worthwhile [22]. The remaining two phantoms were filled only with saline solution so that one had an empty middle part volume and the other was filled. The salinity of the saline solution used in the phantoms was close to that of a normal saline (NaCl 0.9%).

### Fluid conductivities used in phantoms

The four-point conductivity measurement method was used to measure the conductivities of the saline solution and sun flower oil used in the test phantoms. A known alternating current was driven through the volume filled with the fluid under test. Two electrodes with a known distance between them were placed in the fluid and the difference in the voltage between the electrodes was measured at six different frequencies from 100 kHz to 200 kHz in 20-kHz steps. A stand-alone preamplifier was used (Model 5113 PRE-AMP, Signal Recovery, United Kingdom). The electrode cell constant was 25.98 m^-1^.

### Q of the sensor

The quality factor of the tuned coil was measured with an Agilent 4395A network analyser by using an Agilent 43961A RF Impedance Test Adapter. The measurements were made for a coil laid on a table and placed upright on the floor. The interesting bandwidth is from 100 kHz to 200 kHz, but some extra measurements were taken at higher frequencies to give an idea of the trend.

### Driver amplifier stability

The stability of the output voltage of the driver amplifier was measured for every resonance frequency with different coil loadings. The coil was loaded by a loop (*d *= 285 mm) placed in the middle of it. The loop was closed with a resistor whose value varied from 10 Ω to 100 kΩ in one-decade steps.

## Results

### Phantom test

**Figure 7:**
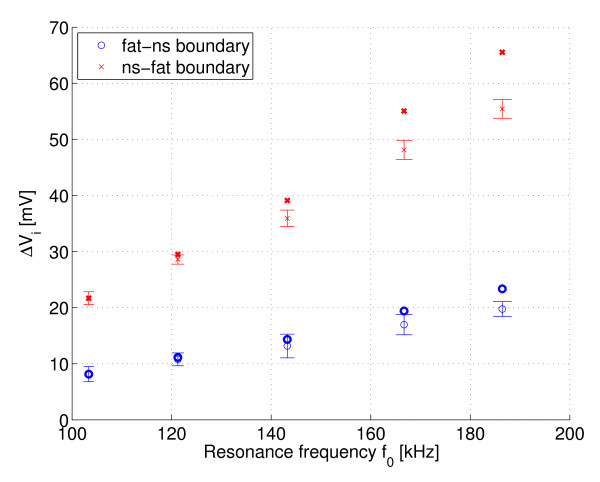
**I channel: Fat-NS and NS-Fat phantoms**. Measured Δ*V*_i _= *V*_loaded _- *V*_empty _(I channel) at different resonant frequencies for phantoms with fat-normal saline boundary and normal saline-fat boundary, n = 10. Points in boldface are the mean values multiplied by the compensation factor k.

**Figure 8:**
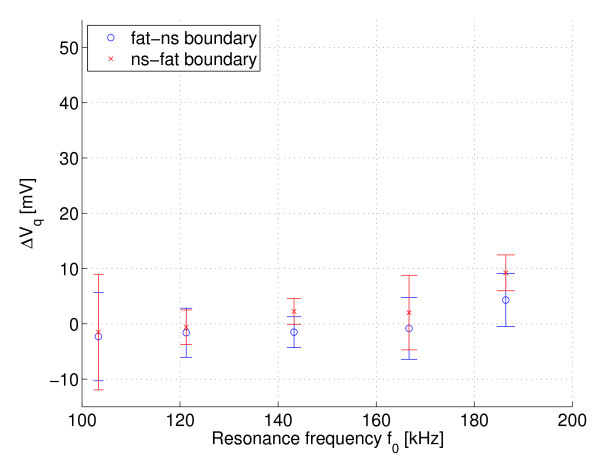
**Q channel: Fat-NS and NS-Fat phantoms**. Measured Δ*V*_q _(Q channel) at different resonant frequencies for phantoms with fat-normal saline boundary and normal saline - fat boundary, n = 10.

**Figure 9:**
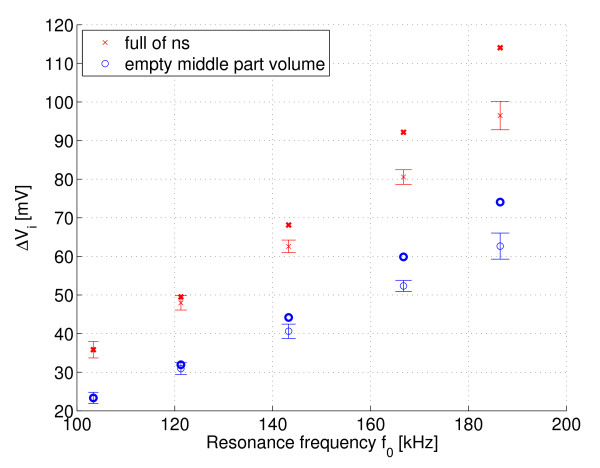
**I channel: air core and full of NS phantoms**. The same measurement as in Figure 7, but now for phantoms filled with normal saline only.

**Figure 10:**
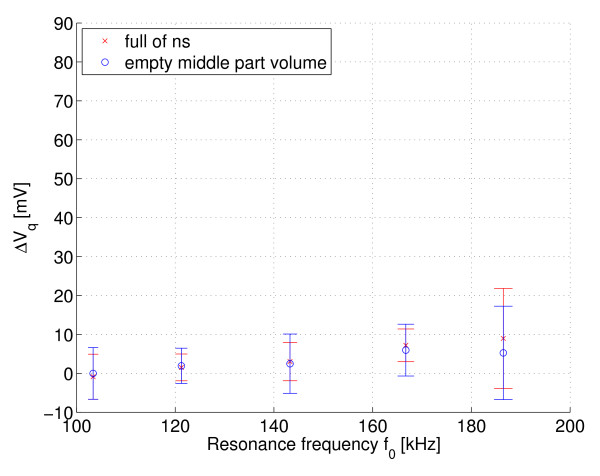
**Q channel: air core and full of NS phantoms**. The same measurement as in Figure 8, but now for phantoms filled with normal saline only.

Figures 7 and 8 show the measured Δ*V*_i _(in-phase) and Δ*V*_q _(quadrature phase) respectively over the resonant circuit when the phantoms with different fat distribution were placed in its coil. Note that the curves are inverted because of the instrumentation used. The error bars are the standard deviations and the points in boldface are the mean values multiplied by the compensation factor *k *≈ *Q*_1_*f*_2_/*Q*_2_*f*_1_. Figures 9 and 10 show the same measurement for the remaining two phantoms filled only with saline solution. The standard deviation of the resonance frequencies *f*_0 _was 1.65-5.5 Hz, depending on the frequency and phantom.

To estimate the electrical loss distribution between the inner and outer parts of these measured phantoms, it is worthwhile to calculate the signal slopes for in-phase signals. The signal slopes for the fat-ns and ns-fat phantoms are approximately 0.14 mV/kHz and 0.41 mV/kHz, respectively. The signal slopes for the partially filled (air core) and entirely filled phantoms are approximately 0.47 mV/kHz and 0.73 mV/kHz, respectively. The difference between the samples and the estimated function values of linear fit (residuals) is below ± 2 mV.

### Fluid conductivities used in phantoms

**Figure 11:**
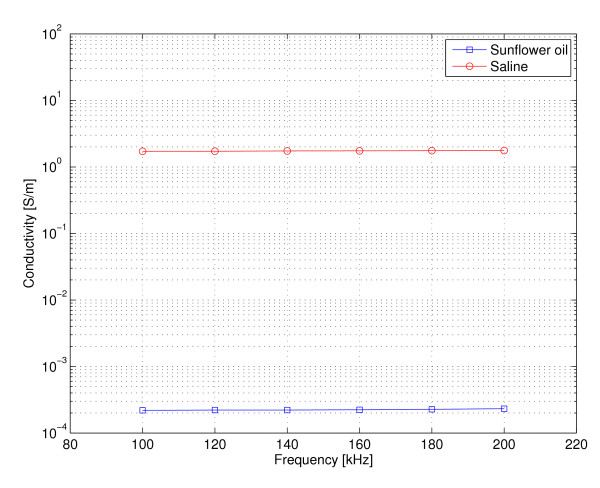
**Fluid conductivities**. The measured fluid conductivities used in test phantoms through the BELA bandwidth from 100 kHz to 200 kHz. The four-point conductivity measurement method was used.

The measured conductivities for saline solution and sun flower oil through the BELA bandwidth from 100 kHz to 200 kHz are shown in Figure 11.

### Q of the sensor

**Figure 12:**
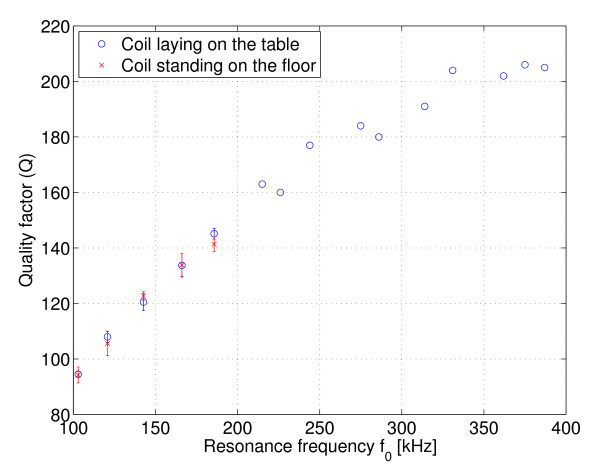
**Q of the resonator**. Quality factor characteristic of the resonator from 100 kHz to 400 kHz.

The quality factor characteristic of the tuned coil is shown in Figure 12. There was no significant change in *Q *over the bandwidth of interest when the coil was laid on a table or placed upright on the floor.

### Driver amplifier stability

The stability of the amplifier output voltage V_0 _was approximately ± 1 mV for the various coil loadings. To ensure that electromagnetic interference did not play a part, the spectrum of the coil was measured from 50 kHz to 450 kHz. The highest peak of 38 dB *μ*V was observed at 66 kHz.

## Discussion

The results of this study show that the BELA method has the potential to be used in the assessment of body composition. It meets the requirement of a device suitable for medical check-ups and repeated individual use. It is non-invasive, easily transferable, fast, and inexpensive compared to tomography. The maximum current flowing in the coil is approximately 4-7 mA, depending on the resonance frequency. From this current the peak value of the magnetic field in the coil was estimated to be approximately 2.2 *μ*T. This is well below the ICNIRP guidelines (International Commission on Non-Ionizing Radiation Protection) for general public exposure, which is 4.6 *μ*T at a frequency of 200 kHz.

By analysing the results of the phantom test, the ratio of the signal slopes between ns-fat and fat-ns phantoms is approximately 2.9. This result is consistent with what was expected. The rate of loss as a function of frequency is higher for the phantom which has saline solution in its outer shell volume and fat in its middle part volume. The magnitude of the signal from the phantom entirely filled with saline solution is about 1.5 times higher than the magnitude of the signal observed from the partially filled (air core) phantom. This indicates a good penetration of magnetic field into the phantom. The magnitude of the signal slope for the phantom with an air core resembles that of the ns-fat phantom, but is slightly higher because air is an even better insulator than fat.

The change in channel Q indicates that there is no significant unwanted capacitive coupling between the coil and phantom below 150 kHz. The mean value of the signal is almost zero. However, at higher frequencies the mean value has slightly increased. A small dielectric loss may have been included as a result of capacitive coupling (recall inverted curves), although the detuning caused by the phantom was insignificant. An electrostatic shield was not used in this experiment, but it is generally essential: see, for example, [6].

The electrical loss resistances of 0.4-5.6 mΩ observed in this study can be compared to MRI studies. An experimental value of a 30 mΩ loss resistance was obtained for a 2-litre saline sample with an 100 mM concentration and *ω*_0 _= 4 MHz, *a *= 0.13 m [23]. By accounting for the factor of 20 in the difference of frequencies, it can be said that the loss resistances match those in this work.

Multiple frequency measurements as made in the BELA method have benefits in probing the conductivity distribution of the subject, by weighting outer structural components as the frequency increases. In body composition analysis this can be used in the assessment of fat distribution, where the single frequency measurement gives only the total conductivity of the subject, and thus indexes such as fat free mass and body water. An example use case for the BELA method can be the measurement of intra-abdominal (IA) fat, as the authors expect that the loss changing rate over frequency will be smaller in an individual who do not have significant amount of IA fat, than in an individual who has. The effect of the subcutaneous fat to the loss changing rate is expected to be minimal. Further studies are required to evaluate this in practice.

## Conclusions

The feasibility of a new electromagnetic method for assessing body composition was introduced. The potential of the method was demonstrated with phantom tests. The results indicate that using different excitation frequencies, it will be possible to estimate the electrical loss distribution between the inner and outer parts of a sample consisting of two differently conductive volumes.

## Competing interests

The authors declare that they have no competing interests.

## Authors' contributions

KB carried out the electronics engineering, performed the sensor analysis, took the measurements and drafted the manuscript. RS participated in the design of the study and revised the manuscript critically. Both authors have read and approved the final manuscript.
